# Magnetic Enhancement of Stem Cell–Targeted Delivery into the Brain Following MR-Guided Focused Ultrasound for Opening the Blood–Brain Barrier

**DOI:** 10.1177/0963689717715824

**Published:** 2017-06-30

**Authors:** Wei-Bin Shen, Pavlos Anastasiadis, Ben Nguyen, Deborah Yarnell, Paul J. Yarowsky, Victor Frenkel, Paul S. Fishman

**Affiliations:** 1 Department of Pharmacology, University of Maryland School of Medicine, Baltimore, MD, USA; 2 Department of Diagnostic Radiology and Nuclear Medicine, University of Maryland School of Medicine, Baltimore, MD, USA; 3 Neurology Service, VA Maryland Healthcare System, Baltimore, MD, USA; 4 Research Service, VA Maryland Healthcare System, Baltimore, MD, USA; 5 Marlene and Stewart Greenebaum Comprehensive Cancer Center, University of Maryland School of Medicine, Baltimore, MD, USA; 6 Department of Neurology, University of Maryland School of Medicine, Baltimore, MD, USA

**Keywords:** blood–brain barrier, magnetic resonance imaging, focused ultrasound, human neural progenitor cells, magnetic attraction

## Abstract

Focused ultrasound (FUS)-mediated blood–brain barrier disruption (BBBD) can enable even large therapeutics such as stem cells to enter the brain from the bloodstream. However, the efficiency is relatively low. Our previous study showed that human neural progenitor cells (hNPCs) loaded with superparamagnetic iron oxide nanoparticles (SPIONs) in culture were attracted by an external magnetic field. In vivo, enhanced brain retention was observed near a magnet mounted on the skull in a rat model of traumatic brain injury, where BBBD also occurs. The goal of the current study was to determine whether magnetic attraction of SPION-loaded hNPCs would also enhance their retention in the brain after FUS-mediated BBBD. A small animal magnetic resonance imaging (MRI)-guided FUS system operating at 1.5 MHz was used to treat rats (∼120 g) without tissue damage or hemorrhage. Evidence of successful BBBD was validated with both radiologic enhancement of gadolinium on postsonication TI MRI and whole brain section visualization of Evans blue dye. The procedure was then combined with the application of a powerful magnet to the head directly after intravenous injection of the hNPCs. Validation of cells within the brain was performed by staining with Perls’ Prussian blue for iron and by immunohistochemistry with a human-specific antigen. By injecting equal numbers of iron oxide (SPIONs) and noniron oxide nanoparticles–loaded hNPCs, each labeled with a different fluorophore, we found significantly greater numbers of SPIONs-loaded cells retained in the brain at the site of BBBD as compared to noniron loaded cells. This result was most pronounced in regions of the brain closest to the skull (dorsal cortex) in proximity to the magnet surface. A more powerful magnet and a Halbach magnetic array resulted in more effective retention of SPION-labeled cells in even deeper brain regions such as the striatum and ventral cortex. There, up to 90% of hNPCs observed contained SPIONs compared to 60% to 70% with the less powerful magnet. Fewer cells were observed at 24 h posttreatment compared to 2 h (primarily in the dorsal cortex). These results demonstrate that magnetic attraction can substantially enhance the retention of stem cells after FUS-mediated BBBD. This procedure could provide a safer and less invasive approach for delivering stem cells to the brain, compared to direct intracranial injections, substantially reducing the risk of bleeding and infection.

## Introduction

A goal of cell-based therapy for brain injuries and neurodegenerative diseases is to provide replacement of neurons, astrocytes, and oligodendrocytes.^1^ Despite advances in cell delivery procedures, cell transplants in animal models and patients have required direct needle injections into the brain. Not only is this highly invasive method associated with serious risks of bleeding and infection, but its efficacy is frequently limited by the inadequate distribution of the injected cells.^2^ Although the vasculature is an effective route of delivery for many therapies outside of the brain, the blood–brain barrier (BBB) can significantly limit the delivery of cells and subsequently affect the efficacy of cell-based therapies.^3^

Focused ultrasound (FUS) has been shown to safely and noninvasively open the BBB in a local and transient manner.^4^ The FUS-mediated blood–brain barrier disruption (BBBD) procedure, carried out under magnetic resonance imaging (MRI) guidance (magnetic resonance–guided focused ultrasound [MRgFUS]), involves the administration of a microbubble suspension comprised of Food and Drug Administration–approved ultrasound contrast agents prior to the FUS exposure. The varying pressure field of the FUS wave is thought to generate controlled oscillation of the bubbles, which then interact with endothelial cells. These mechanical interactions can transiently compromise the integrity of the endothelial cell tight junctions, rendering the vessels leaky.^5^ Studies in rodent models have shown this procedure to enhance the delivery of chemotherapeutic agents,^6^ monoclonal antibodies,^7^ neurotrophic factors,^8^ and drug-loaded nanoparticles^9^ into the brain without overt pathology. This procedure was also shown to enable the delivery of neural stem cells^10^ and natural killer (NK) cells,^11^ where intracarotid artery (IA) injections were used in the former study and less invasive intravenous (IV) injections in the latter study.

In a study using NK cells, a 5-fold increase in delivery to the brain was found when the MRgFUS exposure was carried out in the presence of cells (e.g., cells were injected prior to the FUS exposures) compared to injections of the cells alone. This remains in contrast to the case when cells were injected after the FUS exposures, where only a 2-fold increase was observed.^11^ These results point to a very short “window” of delivery afforded by the FUS exposures for altering the permeability of the BBB. Even under optimal conditions, where cells were injected prior to the exposures, the ratio of NK cells to metastatic breast tumor cells—the targeted tissue in the study—was only 1:100. In the stem cell study, where human neural progenitor cells (hNPCs) were injected using the more invasive IA injections, comparatively smaller numbers of cells were observed at 24-h posttreatment. The cells, however, were shown to be viable and had even begun to differentiate into neurons.^10^ Together, these studies demonstrate the feasibility of using FUS-mediated BBBD for enhancing cellular delivery to the brain. However, they also point to the need to improve delivery and retention of cells if this procedure is to become a viable and efficacious alternative in the clinic for more invasive transplantation procedures that currently require intracranial surgeries.

Cells grown in culture will engulf particles including superparamagnetic iron oxide nanoparticles (SPIONs). This is the basis for tracking transplanted cells in vivo with MRI.^12–17^ Because SPION-loaded cells will be influenced by a static magnetic field,^14,16^ magnetic attraction has been shown to enhance the delivery and targeting of cells, including stem cells, to the liver,^18,19^ heart,^20^ and muscle^21^ by placing an external magnet over the targeted anatomical region. Cell types in these studies have included endothelial progenitor cells (EPCs),^22^ mesenchymal stem cells (MSCs),^23^ and hNPCs.^14–16^ In our previous work in a rodent model of traumatic brain injury (TBI), we demonstrated that the altered permeability of the BBB improves the delivery of hNPCs and is further enhanced when the SPION-loaded cells are used with the application of an external magnet.^15^

Since the efficiency of either strategy for the delivery of stem cells to the brain is extremely low, the goal of our current work was to test the hypothesis that magnetic attraction could increase the delivery of stem cells to the brain after the BBB has been transiently disrupted using FUS. To date, the only published report on using FUS to open the BBB to improve the delivery of hNPCs into the brain employed direct injections into the carotid artery in rats. In the current study, our intention was to evaluate the ability to use safer and less invasive IV injections of hNPCs. We also loaded the hNPCs with SPIONs and employed the use of a static magnet to potentially improve delivery and retention of the cells in the targeted region. The efficacy of this novel combination treatment was evaluated quantitatively by comparing the targeting of iron oxide and fluorescently labeled cells and control noniron-only fluorescently labeled cells that were injected as an equal mixture. Various magnets were also evaluated in terms of field strength and configuration of the applied magnetic fields.

## Materials and Methods

### Experimental Animals

**Figure 1.**
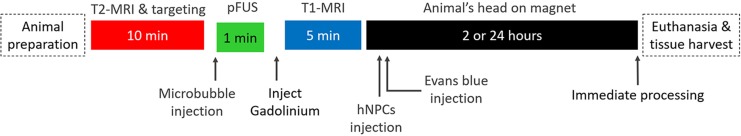
Time line of the experimental procedures used in the study. The specific reagents and magnet types employed are listed in the Materials and Methods section. T2-MRI, T2-magnetic resonance imaging; hNPCs, human neural progenitor cells.

All animal experiments were performed under the guidelines of the institutional animal care and use committee (IACUC), which approved all animal study protocols. All treatments were carried out in female adult Sprague-Dawley rats (100 to 120 g). Animals were housed in pairs in the animal facility with ad libitum access to food and water. All procedures were performed under anesthesia. The experimental procedure time line is summarized in Fig. 1.

### MRgFUS Exposures and Confirmation of BBB Opening

**Figure 2.**
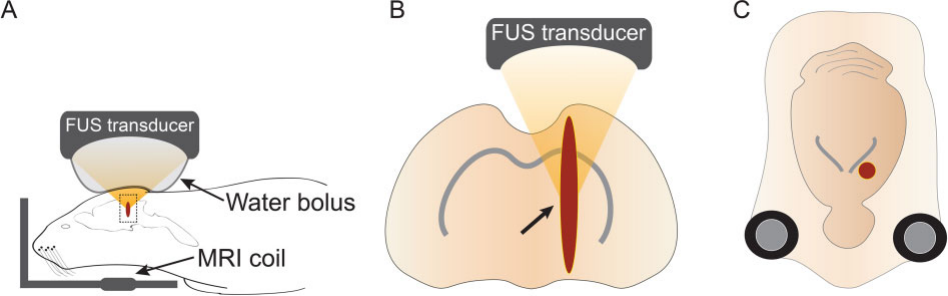
Schematic representation of the focused ultrasound (FUS) exposures. (A) FUS) exposures were provided noninvasively in the brain, where a flexible, acoustically transparent bolus, filled with degassed deionized water and able to deform to the shape of the head, was used to couple the transducer directly to the dorsal region of the head. The head region and the transducer were situated within the magnetic resonance imaging coil inside the magnet bore, which was used for both targeting and monitoring of the exposures. (B) The FUS exposures were targeted at the striatum (arrow), seen in the axial view. However, because of the relatively large size of the long axis of the focal zone of the ultrasound beam to the rat brain, the active region of treatment (dark red) extended from the dorsal cortex to the ventral striatum. (C) Coronal view of the rat brain showing the relative size of the radial diameter of the focal zone. Image not to scale. MRI, magnetic resonance imaging.

All MRgFUS treatments were performed under guidance of a Bruker Biospec 7.4 Tesla small animal MRI scanner (Bruker, Billerica, MA), possessing a 30-cm horizontal bore. The treatments were carried out noninvasively using a commercial MRgFUS system (Image Guided Therapy, Pessac, France), specifically designed to safely provide transcranial FUS exposures in rodents.^24,25^ The MRgFUS system was customized to be compatible with the specific MRI scanner. Prior to the treatments, animals were anesthetized by 2% isoflurane inhalation (Kent Scientific, Torrington, CT) using a benchtop anesthesia chamber with a scavenging system. The animal’s head was shaved to maximize coupling. The animal was then placed in the MRgFUS stage, which is similar to a standard animal MRI stage including an anesthesia nosecone. Once the anesthetized animal was properly secured in the holder and the transducer placed over the animal’s head, the entire assembly was inserted in the bore of the scanner; note that the transducer was coupled to the head with a bolus (e.g., inflated membrane extending from the face of the transducer) containing degassed water (Imasonic, Voray-sur-l’Ognon, France; Fig. 2A). Ultrasound gel was applied to the region of contact between the bolus and the head to further ensure maximum coupling. The animal was kept warm with a heated water jacket, and its respiration rate was monitored remotely, where the level of anesthesia could be varied accordingly. A T2-weighted scan in the region of the treatment was performed prior to the exposures for treatment planning and targeting, similar to the clinical MRgFUS systems.^26^ For each treatment, exposures were carried out at a single location, where the geometric center of the focal zone was targeted at the striatum. Because the axial length of the focal zone was approximately 7 to 8 mm (−3 dB), which is similar to the diameter of the brain, the effective region of treatment extended well into the dorsal cortex and ventral striatum (Fig. 2B). The exposures were provided at 1.5 MHz, in 5 ms bursts, at a pulse repetition frequency of 10 Hz, and 5% duty cycle for a total duration of 60s. The axial view of the treated spot is shown in Fig. 2C. An acoustic pressure of 0.45 MPa was used based on previous studies on the use of FUS for opening the BBB and also carried out at 1.5 MHz.^27,28^

**Figure 3.**
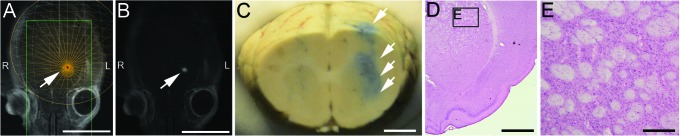
Magnetic resonance–guided focused ultrasound (MRgFUS)-mediated opening of the blood–brain barrier. (A) A T2-weighted, axial MR image from the MRgFUS graphic user interface showing the procedure employed for the exposures. The arrow points to the focal zone of the ultrasound beam. The interface provides also the coronal view, allowing for the targeting to be carried out in each dimension. The scale bar corresponds to 10 mm. (B) A T1-weighted axial image following the focused ultrasound (FUS) exposure and administration of an MR-compatible contrast agent. The arrow points to a hyperintense region, indicating extravasation of the contrast agent at the region of treatment in “A.” The scale bar corresponds to 10 mm. (C) Evans blue dye (arrows) in the left hemisphere of a paraformaldehyde-fixed brain. The dye is found throughout the region of the focal zone of the ultrasound beam. The scale bar corresponds to 2 mm. (D) Representative histological section of the left hemisphere of a FUS-treated brain; the scale bar corresponds to 2 mm. (E) Box from “D” showing higher magnification of the striatum. Normal cytoarchitecture is seen without evidence of damage to the tissue. The scale bar corresponds to 200 µm.

Once the animals were securely placed in the scanner, T2-weighted MRI scans of the entire brain in both the axial and coronal views were captured. The images were transferred electronically to the graphic user interface of the MRgFUS system, and used for targeting, where the position of the transducer and the focus of the ultrasound beam were overlaid on them (Fig. 3A). Immediately prior to the FUS exposure, a suspension of microbubbles was administered IV via a previously inserted catheter in the tail vein. Vials of size-isolated monodisperse microbubbles (SIMB4-5; Advanced Microbubbles Laboratories, Boulder, CO) with diameters between 4 and 5 μ were prepared for use per the manufacturer’s instructions. A 250 μL injection was given at a concentration of 2.27 × 10^9^ microbubbles per mL. Following the FUS exposure, opening of the BBB in the treated region was confirmed on a T1-weighted MRI after tail vein injection of gadodiamide contrast agent at 0.2 mL/kg (Omniscan, GE Healthcare, Princeton, NJ).

### Stem Cell Injection and Static Magnet Exposure

After successful opening of the BBB, indicated by a hyperintense signal following the gadodiamide injection in the region of treatment in the T1-weighted MR images, animals were removed from the scanner. Under anesthesia, administered intraperitoneally, ketamine at 60 mg/kg (Henry Schein, Melville NY) and xylazine at 7.5 mg/kg (Akorn Pharmaceuticals, Lake Forest, IL),^15^ animals were positioned in the supine position, with their head resting on one of the 3 different magnets: a 2″ diameter × 2″ length or 3″ × 3″ neodymium cylinder magnets (K&S Magnets, Pipersville, PA) or a Halbach array magnet (courtesy of Dr. Mark Lythgoe). The Halbach magnet possessed a cylindrical array, with 12 elements of 60 mm in width, with a bore diameter of 8 cm and length of 20 cm.^29^ Animals were kept under a heat lamp to maintain body temperature for all magnet incubations. Injections of the hNPCs were then administered intravenous (IV).

hNPCs used in the study were labeled with SPIONs; more specifically, we used Molday iron oxide nanoparticles (MIONs; EverGreen, BioPal Worcester, MA, USA) and sans iron oxide rhodamine B (SIRBs) particles (rhodamine B) for the cell labeling (BioPal, Worcester, MA) as previously described.^14,16^ The SIRB nanoparticles were similar to the MION nanoparticles except they lacked an iron oxide core. After labeling, MION-hNPCs or SIRB-hNPCs were dissociated, counted, and centrifuged at 85× *g* for 10 min. The MION-hNPCs and SIRB-hNPCs were resuspended in neurobasal medium (ThermoFisher Scientific, Pittsburgh, PA) and mixed in a ratio of 1:1 with a final cell density of 3 × 10^6^ cells/mL. Prior to the hNPC injections, animals were injected IV with sodium nitroprusside (Sigma-Aldrich, St. Louis, MO) at 25 mg/kg diluted in 100-μL phosphate buffered saline (PBS). For all hNPC injections, a total of 1.5 × 10^6^ cells in 0.5 μL of PBS was injected per animal in the presence of the various magnets. Following the stem cell injections, 150 μL of Evan’s blue dye (EBD) at 2% (Sigma-Aldrich, St. Louis, MO) was similarly injected IV.^30^ For all treatments, animals were maintained for 2 h on the magnet. Animals were sacrificed at different time points: immediately following the 2-h magnetic exposure or 24 h (Halbach array only) after injection of hNPCs. Four animals were used for each time point and magnet type.

### Cell Counts and Histological Analysis

Histological analysis of brain sections was performed as previously described.^15^ Briefly, after hNPCs injection and static magnetic exposure, animals were either euthanized followed by the perfusion with 4% paraformaldehyde at 7.4 pH (Sigma-Aldrich, St. Louis, MO) or maintained for 24 h and then euthanized. The brains were extracted and sectioned on a cryostat at 40-μm slices. To visualize SPION-loaded hNPCs, the brain sections were incubated in Perls’ Prussian blue consisting of 2% hydrochloric acid and 2% potassium ferrocyanide (Sigma-Aldrich, St. Louis, MO) staining solution for 10 min at room temperature, followed by counterstaining with Mayer’s Hematoxylin (Sigma-Aldrich, St. Louis, MO), dehydration, and placement of a coverslip. To quantitatively analyze the effects of static magnet attraction on SPION-loaded hNPCs, both MION-hNPCs and SIRB-hNPCs (total cells) were counted at 1-mm regions from the dorsal to the ventral cortex. The distribution of both cell types was characterized over these regions. The ratio of MION-hNPCs and SIRB-hNPCs was determined in both the dorsal and ventral cortex for comparison. The statistical significance of the intergroup differences was assessed using the Student’s two-tail *t-*test (Microsoft Excel, Redmond, WA). The mean ± standard deviation (*SD*) was determined. Statistical significance was determined with a *P* value of less than 0.05. To confirm that MION-hNPCs were human progenitor cells, the brain sections were labeled with SC121 antibody, recognizing human-specific cytoplasmic antigen (Stem Cells Inc., Cambridge, UK).^16^ Fluorescent microscopic images were collected using a Zeiss Axio Observer Z1 inverted microscope (Carl Zeiss, Jena, Germany). Prussian blue-stained images were collected using a Nikon Eclipse 80i microscope (Nikon, Tokyo, Japan).

## Results

**Figure 4.**
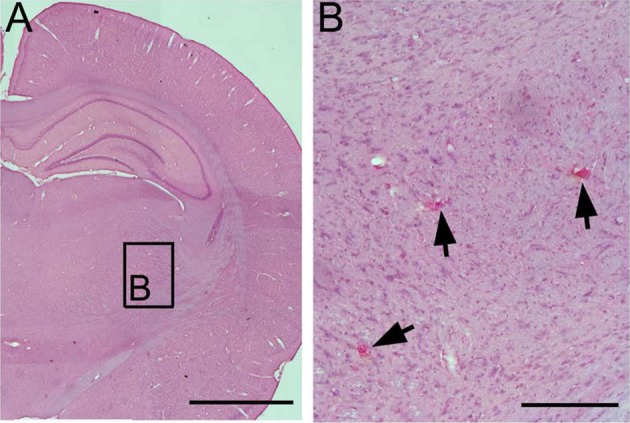
Tissue damage at higher amplitude focused ultrasound (FUS) exposures for blood–brain barrier disruption (BBBD). FUS exposures carried out at 0.75 MPa (as opposed to 0.45 MPa for the remainder of the study) were shown to cause localized red blood cell extravasation (arrowheads) in the midbrain (mesencephalic) regions. (A) Micrograph of hematoxylin and eosin brain section at a magnification of 2×. The scale bar corresponds to 2 mm. (B) Box from “A” depicting a higher magnification at 20×. The scale bar corresponds to 200 µm.

The FUS exposures were found to be successful for opening the BBB using the treatment procedure described for the study. Following the administration of gadiodiamide contrast, T1-weighted MR images showed hyperintense signals at the targeted region (Fig. 3A and B), evidence of localized enhancement of BBB permeability. These signals were observed at the region being targeted (e.g., the striatum). Additionally, following FUS, EBD was visualized in fixed whole brains, where the presence of the dye extended outward from the striatum to the dorsal cortical regions of the brain (Fig. 3C). Histological sections stained with hematoxylin and eosin did not show evidence of damage to the tissue incurred from the FUS exposures (Fig. 3D and E). When using a higher pressure amplitude of 0.75 MPa (as opposed to 0.45 MPa), opening of the BBB was also observed in postcontrast, T1-weighted images. However, for these exposures, evidence of hemorrhage was found in the form of small, localized regions of red blood cell (RBC) extravasation (Fig. 4).

**Figure 5.**
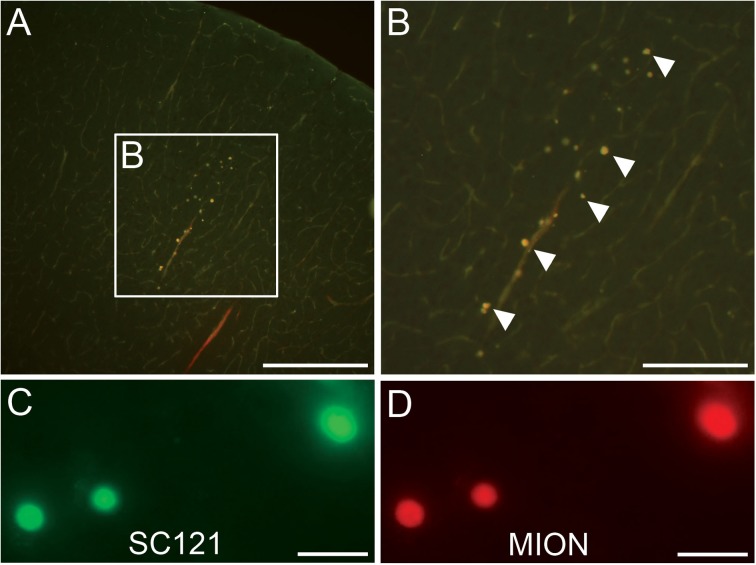
Cortical location of transplanted superparamagnetic iron oxide nanoparticles engulfed in human neural progenitor cells (SPION-hNPCs) following blood–brain barrier disruption (BBBD) and magnetic attraction. (A) SPION-hNPCs are found in the dorsal cortex. The scale bar corresponds to 400 μm. (B) The magnified region as indicated in the previous panel “A.” The scale bar corresponds to 200 μm. (C) These cells are immunopositive for SC121 (the human cytoplasmic antigen) and constrain the fluorescent dye linked to the nanoparticle Molday iron oxide nanoparticles. The scale bar corresponds to 20 μm. (D) C121 positive cells constraining the fluorescent dye linked to sans iron oxide rhodamine B particles. The scale bar corresponds to 20 μm.

**Figure 6.**
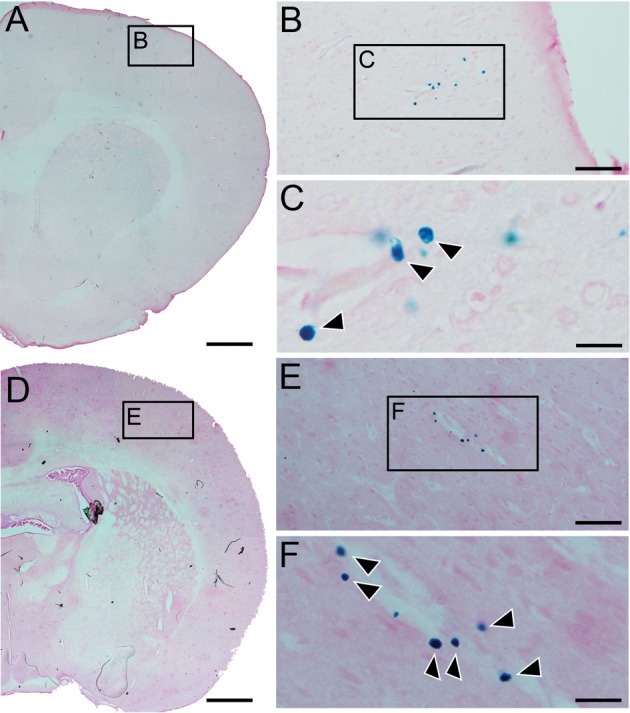
Molday iron oxide nanoparticles (MION)-labeled cells in the focused ultrasound (FUS)-treated tissue. (A) Stem cells present within the focal zone of the blood–brain barrier disruption (BBBD) region in the dorsomedial cortex 2-h post-FUS exposure. The scale bar corresponds to 1 mm. (B) Transplanted MION-labeled cells were identified using Perl’s staining. The scale bar corresponds to 50 µm. (C) Box in “B” showing MION-labeled cells at a higher magnification (arrowheads; 40×). The scale bar corresponds to 5 µm. (D) Stem cells present within the focal zone of the BBBD in the dorsomedial cortex after 24-h post-FUS exposure. The scale bar corresponds to 1 mm. (E) Transplanted MION-labeled cells 24-h post-FUS were identified using Perl’s staining. The scale bar corresponds to 50 µm. (F) Box in “E” showing MION-labeled cells at a higher magnification (arrowheads; 40×). The scale bar corresponds to 5 µm.

At 2-hr postinjection, iron oxide labeled hNPCs were identified in the brain sections of animals receiving FUS exposures, followed by IV administration of cells in the presence of a static magnet (Fig. 5A and B). Additional validation of the presence of the cells was performed using immunohistochemistry, where the fluorescent signals of the MION-hNPCs (Fig. 5C) colocalized with those of a fluorescent-labeled antibody for the SC121 positive human-specific antigen (Fig. 5D). SPION-loaded cells were observed only in the FUS-treated region by Perls’ Prussian blue (Fig. 6A to C). Perls’ Prussian blue–labeled stem cells were also observed at 24-h postinjection, however, to a lesser degree (Fig. 6D to F).

**Figure 7.**
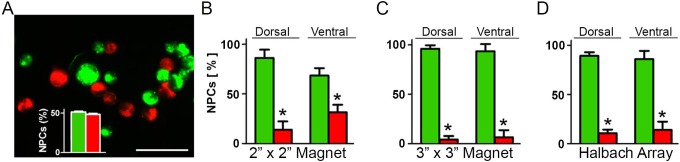
Quantifying human neural progenitor cells (hNPCs) in the brain. (A) A representative fluorescence microscopy image showing both Molday iron-oxide nanoparticles (MIONs) within hNPCs (EverGreen) and superparamagnetic sans iron-oxide nanoparticles (SIRB) within hNPCs (rhodamine B). Inset shows the relative percentage of each cell type employed for the injections. The scale bar corresponds to 50 µm. (B–D) Relative percentages of MION-hNPCs (EverGreen) and SIRB-hNPCs (rhodamine B) in the dorsal (cerebral cortex) and ventral (striatum) brain regions when employing the 3 different magnets. The plot bars represent ±standard deviation from 3 rats with a total of 279 cells counted for “A,” 3 animals with 223 cells counted for “B,” and from 2 rats where 345 cells were counted for “C.” *Indicates a difference between MION- and SIRB-labeled cells at a level of significance less than 0.05.

**Figure 8.**
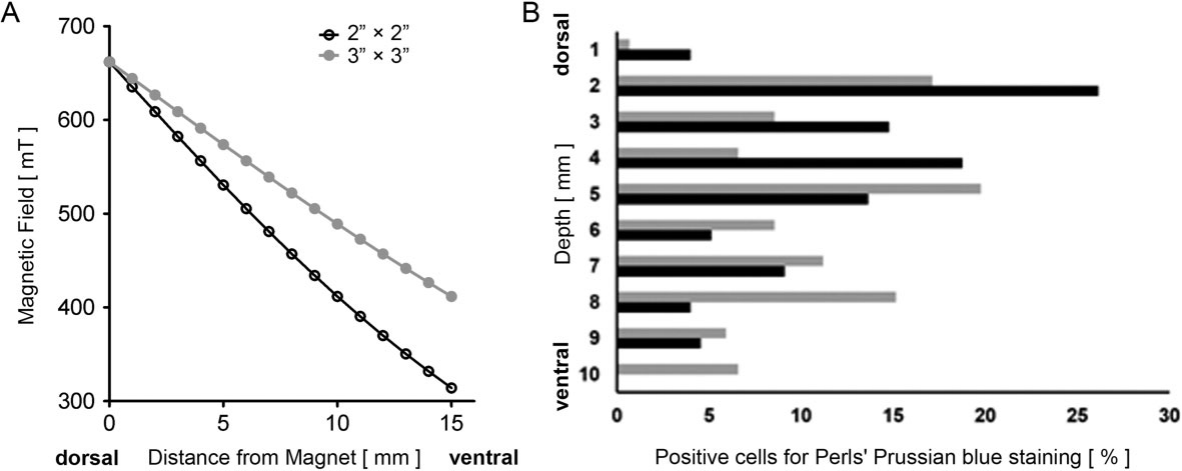
Magnetic flux density and depth associated human neural progenitor cells (hNPCs). (A) Magnetic flux density profiles for the 2″ × 2″ and 3″ × 3″ magnets. (B) The percentage of total hNPCs in relation to depth within the treated brain where 1 mm is the outer region of the dorsal cortex (closest to the magnet) and 10 mm is the outer region of the striatum (farthest from the magnet). Representative data are presented for each magnet.

To directly determine the effect of magnetic enhancement of stem cell–targeted delivery, we injected a mixture of cells loaded with SPION (MION, EverGreen) with an equal number of cells loaded with a similar size, but nonmagnetic nanoparticle (SIRB, rhodamine D) that served as a nanoparticle control^15^ (Fig. 7A). In treatments that employed the 2″ × 2″ magnet, a majority of the fluorescent MION-hNPCs were found in the sampled brain region of the dorsal cerebral cortex, located close to the skull and surface of the external magnet. Here, 86% of the total fluorescent hNPCs were MION-hNPCs. In deeper, striatal regions in the brain less overall cells were observed, where only 68% of the fluorescent cells were MION-hNPCs (Fig. 7B). When using the 3″ × 3″ magnet, the percentage of MION-hNPCs to that of the total number of cells was 96% and 93% in the dorsal and ventral brain, respectively (Fig. 7C). With the Halbach array, the percentage of MION-hNPCs in the dorsal and ventral brain was 89% and 86%, respectively (Fig. 7D). Total cell counts of iron-containing cells in the sonicated regions were obtained from Perls’ stained sections with 224 and 133 for the 2″ × 2″ magnet; 274, 426, and 140 for the Halbach array; and 117 for the single animal for the 3″ × 3″ magnet. For all magnet types, and in both the dorsal and ventral regions, the percentage of MION-hNPCs was significantly greater than that of SIRB-hNPCs. Furthermore, the trend of less overall cells in the striatum compared to the dorsal cortex was maintained for all 3 magnet types. The gradient in the numbers of cells, going from dorsal to ventral regions, was also steeper using the 2″ × 2″ magnet compared to the 3″ × 3″ magnet and the Halbach array (Fig. 8). The Halbach array was the only magnet used for the 24-h time point. Here, less overall cells were observed than at 2 h, and these were found to be mostly MION-hNPCs and found exclusively in the dorsal cortex.

## Discussion

In the current study, we demonstrated how combining FUS exposures for opening the BBB and magnetic targeting can enhance the local delivery of hNPCs to the brain compared to the FUS exposures on their own. Many studies have shown how FUS-mediated BBBD can improve the local delivery of a wide variety of agents,^6–9^ although attempts to deliver cells, the largest form of potential therapy to the brain with this strategy is extremely limited.^10,11^ The use of magnetic targeting has been predominantly restricted to tissues outside the brain because of the BBB. Experience with the delivery of cells to the brain with magnetic targeting is also extremely limited and has been restricted to settings where the BBB has been compromised such as stroke and trauma.^15,23,31,32^ In our previous study, magnetic targeting was able to improve retention of SPION-labeled cells in the traumatized region in a rat TBI model.^15^ It is somewhat surprising that combining FUS with magnetic targeting has not yet been well explored. They are clearly compatible methods, where in our current study, the external magnet was put in place directly after the animal was removed from the MRI, while others have even proposed using the MRI itself to create the targeting field.^33,34^ Both of these minimally invasive methods suffer from poor efficiency of delivery, particularly for larger therapeutics such as cells. In the only previous study of stem cell delivery using FUS to disrupt the BBB, only 33 cells/mm^2^ per section area were found within the sonicated region in spite of direct intracarotid injection.^10^ We calculate from this publication that the approximate total number of cells in the region of sonication was approximately 500 cells or 0.025% of the cells injected. In the rare studies in which cell number was quantitated, the fraction of injected cells retained in the brain after magnetic targeting was also very small. Li et al.^31^ noted that after an IV injection of 10^6^ endothelial cells labeled with SPIONs, the application of a magnetic field increased the number of cells within the target infarction region 3-fold. Although this change is impressive, the absolute number of cells remained small, increasing from 25 to 75 cells per section.^31^ Only 2 previous studies combining ultrasound and magnetic targeting have been reported for enhancing delivery. In one study in a flank tumor model, magnetic targeting was used to localize the delivery of microbubbles for ultrasound-mediated release of a fluorophore loaded in the microbubbles.^35^ Another study demonstrated how superparamagnetic iron oxide–doxorubicin microbubble complexes could be used to enhance the delivery of the drug in a rat glioma model.^36^ Our current study is the first to report how ultrasound and magnetic targeting can be combined to improve delivery of cells to the brain.

The FUS exposures used in this study, especially the acoustic pressure, were based on those previously described for opening the BBB using a device similar to ours.^24,25^ Keeping all other exposure parameters constant, we evaluated additional exposures that possessed higher acoustic pressures. The acoustic pressure, specifically the peak negative pressure, is the primary factor for activating the microbubbles, allowing them to transition to a state of stable oscillation, leading to opening of the BBB.^37^ The reigning hypothesis of how FUS exposures combined with the systemic administration of microbubbles can alter BBB permeability states that as the bubbles oscillate within a blood vessel, they generate a host of nondestructive, mechanical interactions with the vessel wall, including the generation of microstreaming and direct physical interactions. These ultimately render the vessels permeable, presumably by compromising the integrity of the tight junctions between individual endothelial cells.^38,39^ By increasing the acoustic pressure from 0.45 MPa to 0.60, 0.75, and 0.90 MPa, we found we could also open the BBB. As demonstrated previously,^40^ however, we did observe evidence of unwanted structural alterations in the tissue in the form of RBC extravasation when using these higher pressures. The degree of these effects was notably proportional to the acoustic pressure of the exposures, as previously reported.^40^ Typically, as the acoustic pressure increases, stable bubble oscillations (i.e., noninertial cavitation) will become unstable, generating inertial cavitation, where ensuing bubble collapse may generate effects such as blood vessel disruption, leading to unwanted RBC extravasation.^41^

The time points in this study were chosen because of their similarity to the work of Burgess et al.,^10^ the only previous study to deliver stem cells to the brain after disrupting the BBB with FUS, and literature applying a magnetic field, where application of the magnet has ranged from 15 min to several days. Our previous study used a surgically fixed and much smaller magnet which remained in place for 5 days. From the work of Alkins et al.,^11^ which we discussed in the Introduction section, it is clear that BBBD with regard to cell entry is very brief, the rationale for the use of a much larger and more powerful magnet for a much shorter duration of field application. The 2-h end point represents a compromise between the duration of magnetic field application and the duration of time the BBB may still be open to objects as large as cells. A 24-h time point has been frequently used in the literature to assess retention of cells at a time the BBB has likely been reestablished.

The hNPCs, both SPION-loaded and nonloaded, were observed from the outer regions of the dorsal cortex (proximal to the ultrasound transducer) to the outer regions of ventral brain regions (striatum), essentially extending from one side of the brain to the other in the coronal plane. Extravasation of the EBD was similarly found throughout this region. This extent of BBB opening occurred even though the targeted region was the striatum. These results are, however, not surprising, seeing that the long axis of the focal zone for the device being used was approximately 8 mm. Similar results were reported in a study, also in a rat model, that employed FUS for opening the BBB to deliver a chemotherapeutic agent to gliomas. Here, Evans blue dye was also observed from the dorsal to ventral cortex.^36^ That a single exposure could effectively treat such an extensive region is an artifact of the experimental setup and model. In clinical devices that employ multielement ultrasound arrays similar to the one used in the current study, the focal zone is comparable in size. However, the relative size of the focal zone to the size of the human brain in these devices (as opposed to a rat brain used in the current study) is by several orders of magnitude smaller.^42^ The latter also applies in the case of tissues and organs other than the brain (e.g., primary and metastatic tumors) currently being treated with FUS.^43^

In this study, we evaluated two different static magnets with differing diameters to provide some preliminary evidence of the effects of magnetic field strength on the magnetic targeting process. Immediately after the BBB opening procedure, the animals were maintained in the supine position with the dorsal region of their heads in direct contact with the magnet being evaluated. This procedure was deemed suitable for a 2-h magnetic exposure period. This approach is in comparison to our previous TBI study, where a magnetic “hat” was adhered to the head of the animals in order to evaluate the long-term effects (e.g., days) of magnetic targeting.^15^ Adhering magnets to the head of animals for the purpose of targeting has also been used for improving the delivery of nanoparticles to the brain for periods as short as 3 h.^36^ We felt that for our procedure, involving only 2-h exposures with the magnet would be logistically simpler and more consistent to lay the animals’ heads on the magnet. This procedure also allowed us to evaluate and compare different types of magnets as well as those with higher field strengths.

The current study employed a novel experimental approach, where SPION- and non-SPION-loaded cells were labeled with 2 different colored fluorophores, allowing us to clearly distinguish between the 2 different cell populations in sections of the brain, and hence the effects that the magnets produced. This approach was deemed more efficient than traditional studies on magnetic targeting, where typically all cells are labeled with a magnetic compound and 2 different experimental groups are required (in addition to a control), where one employs a magnet and the other involves the administration of cells only.^20,44–46^ Furthermore, by using a combination of SPION-loaded and nonloaded cells, administered in equal amounts, we could evaluate the relative numbers of cells of each type, controlled within the same animal.

The benefits of adding magnetic attraction to the delivery procedure were demonstrated unequivocally for all experimental groups and in all regions treated. Even in the ventral cortical region (striatum), farthest away from the magnet, and when using the least powerful 2″ × 2″ magnet, approximately two-thirds of the hNPCs observed were those loaded with the SPIONs, which enabled them to be influenced by the static magnetic fields.^14–16^ The relative number of observed SPION-loaded cells increased in the dorsal cortex with this magnet to approximately 80%. These results are not surprising, seeing the relatively steep gradient in magnetic flux density with this magnet, where the value decreased approximately 40% from the dorsal to ventral regions within the brain. With the 3" × 3", there was only a 25% drop in magnetic flux density over this same region. This would explain why when employing this more powerful magnet not only was there a greater ratio of SPION-loaded to nonloaded cells observed in the dorsal cortex. But also, why this ratio barely decreased for cells in the ventral region. Indeed, when looking at how the cells distributed throughout the brain from the dorsal to ventral, a steeper gradient in decline was observed for the less powerful 2" × 2 magnet. This gradient in cells was, not surprisingly, less sharp when using the 3″ × 3″ magnet.

Cell-based therapy using magnetic attraction of SPION-labeled stem cells is actively being investigated in ischemic myocardial repair models to enhance the engraftment of cells. Recent studies have shown how this procedure can enhance therapeutic benefits in terms of attenuated left ventricular remodeling and higher injection fractions.^20,45^ A variety of cell types have been evaluated in these studies including MSCs,^46^ EPCs,^44^ and cardiosphere-derived cells.^20,45^ In one study, the effect of magnetic field strength on cellular engraftment was demonstrated, where SPION-loaded MSCs were injected into the left ventricle, and a magnet was placed over the region of injured myocardium. Three weeks after the cells were infused, engraftment rates were 1.88- and 3.06-fold greater using a 0.15- and 0.3 T magnet, respectively, compared to a 0 T magnet.^46^ In the study using EPCs, approximately 10 times the number of cells were found to be engrafted with a 1.5 T magnet compared to not using the magnet.^44^ This ratio is similar to our observations in the dorsal cortex when using the 3″ × 3″ magnet, where between 80% and 90% of the transplanted hNPCs were those loaded with the SPIONs.

We also evaluated an additional magnet known as a Halbach array. The configuration of this magnet involves the arrangement of permanent magnets such that the magnetic field is enhanced on one side of the array and canceled on the other side to almost 0 T.^47^ Cylindrical Halbach arrays, such as the one employed in our study, have been evaluated in in vitro experiments, for example, demonstrating the ability to separate and retain microbubbles in a constant flow that were loaded with magnetic nanoparticles.^48^ Halbach arrays were also employed with SPION-loaded fibroblasts to enhance the rate and uniformity in the seeding process of the cells onto the luminal surfaces of large tubular constructs.^49^ Cylindrical Halbach arrays are currently being proposed for iron-loaded cells for magnetic-targeted delivery for the treatment of peripheral arterial disease.^29^

Our interest in evaluating the Halbach array derives from the fact that the decrease in magnetic flux and attractive force with distance from its surface is typically less steep than a single magnet such as the cylindrical magnets that we also evaluated. The magnetic flux and attractive force for cylindrical magnets were however greater at the surface than for the Halbach array. We therefore expected to obtain more uniform distributions of cells with the Halbach array than with the cylindrical magnets. In our study, the head of the animal was placed within the cylinder. However, the results obtained were not observed to be different than those with the 3″ × 3″ magnet. We are currently working with the manufacture of the array to develop a more powerful and efficient array specifically for our investigations and procedures being developed.

One question that arises from this study is whether the applied magnetic field directed the cells to the targeted region while still in circulation. Or perhaps it only worked to keep the cells in the region where they had extravasated from the vasculature. The experimental design of the study did not allow for this to be determined. Another question is whether other factors were involved in the process that contributed to improving the homing and retention of the cells (both SPION-labeled and none labeled) to the targeted region. Delivery of large therapeutics across the BBB is normally inefficient, where accumulation of 1% to 2% of the total injected agents into the brain is considered to be high.^50^ Even when using FUS for opening the BBB, studies involving molecular or cellular therapies typically find that less than 0.1% of the injected agent/cell can be detected in the sonicated region of the brain.^7,10^ In our previous work, in both skeletal muscle^51^ and kidney^12^ models, we used FUS exposures delivered in pulsed mode (similar to the current study), however, at higher rates of energy deposition. We found we were able to increase the homing and retention of bone marrow–derived stromal cells and mesenchymal stromal cells (MSCs) in the kidneys^12^ and skeletal muscle,^51^ respectively. The underlying mechanism behind these results was presumed to be associated with the observed increases in the levels of cytokines, chemokines, trophic factors, and cell adhesion molecules in the treated tissue, which occurred apparently by the process of mechanotransduction.^52^ Whether this mechanism contributed to improving the targeting of the hNPCs in the current study will also have to be investigated.

### Study Limitations

In the current study, we demonstrated how magnetic targeting and FUS for opening the BBB could improve delivery and retention of hNPCs in the brain compared to FUS alone. There were however several limitations to the study. In this study, we did not separately evaluate the effects of the FUS alone on delivery of nonlabeled hNPCs to the brain, although we found very few SIRB-labeled hNPCs in the same regions as MION-labeled hNPCs. We feel however that this might have been redundant, seeing that earlier studies on cell delivery to the brain via systemic delivery have shown that indeed very few cells can be delivered without the altered permeability of the BBB. This was shown in the case of TBI^15^ or when using FUS for transiently opening the BBB.^10,11^

Furthermore, we did not evaluate the viability of the cells that were delivered to the brain. In our previous study, using magnetic targeting in a model of TBI, we used immunolabeling and Fluoro-Jade C staining to confirm that the hNPCs were indeed viable posttransplantation.^15^ We also demonstrated that labeling of the hNPCs with SPIONs, itself, does not affect their viability or their ability to proliferate and differentiate.^16^ Both these studies further demonstrated a lack of effect of a static magnetic field on the SPION-labeled hNPCs.^15,16^ In a separate study, neural stem cells were similarly delivered to the brain using FUS, though without magnetic attraction. Posttransplantation analysis showed the cells expressed the known stem markers polysialic acid and nestin, which suggests that they remained viable through the transplantation process. The cells also expressed the microtubule-associated protein, doublecortin, which indicated that they maintained the ability to differentiate in to neurons.^10^ We are presently evaluating the procedure described here in a therapeutic model of Parkinson’s disease. Viability assessments of the cells, as well those for proliferation and differentiation, will be carried out. Important, too, in this study will be determining how many cells persist for extended periods of time in the targeted regions. This will have equally important implications for the therapeutic efficacy of the treatments as the viability and functionally of the cells. In our study, we did find cells in the targeted brain tissue at 24 h, albeit in lesser numbers and only in brain regions closer to the magnetic source. These results are still encouraging; however, they further point to the need for additional optimization of the procedure being developed.

As noted earlier, cellular delivery to the brain from the bloodstream may be the most problematic form of therapeutic delivery. Our study was not designed or powered to compare the total number of cells delivered to brain in each experimental group. The average numbers of iron-containing cells we detected in the sonicated region were 224 ± 119. This small number represented 0.03% of the 0.75 × 10^6^ total number of iron-containing cells. This small number is similar (0.025%) to the previous study with hNPCs by Burgess, which used FUS alone but injected cells via intracarotid injection. Successful translation of this strategy to functional benefit in animals or clinical benefit will clearly depend on the absolute number and regional brain density of the transplanted cells. The efficiency of this form of delivery clearly must continue to improve before the minimally invasive methods such as the strategies we present can fulfill the goal of successful clinical translation. Our study and previous literature do provide several paths toward increased the efficiency of stem cell delivery. Although intracarotid infusion is less preferable, it is more efficient that IV injection and far less invasive than intracerebral injection. Newer magnet designs can also be employed with the capacity to create a more powerful magnetic field at a greater depth than our current Halbach array. Stem cells with a greater propensity for chemoattraction and homing such as MSCs may be a more suitable cell type for this method than hNPCs. Our current study represents only one important step in advancing the goal of developing clinically useful strategies for delivery of stem cells form blood to brain.

## Conclusion

Our work preceding the current study demonstrated how labeling hNPCs with SPION does not adversely affect the cells, including when they are exposed to a static magnetic field. We also demonstrated that applying a magnetic field in a model of TBI could improve the retention of the SPION-labeled hNPCs at the sites of altered BBB permeability when compared to the absence of the magnetic field. In the current study, we hypothesized that magnetic attraction of SPION-labeled hNPCs could be used more generally as a procedure for enhancing cellular delivery to the brain that possessed an intact BBB. For this, we employed FUS to selectively open the BBB at a predetermined location where a small number of recent studies had shown proof of concept for safely delivering cells to the brain, albeit in limited quantities. The experimental design we employed allowed us to demonstrate unequivocally that magnetic attraction could significantly enhance the retention of cells in a localized manner at the site of BBB opening, where the degree of enhancement was dependent on the strength of the magnetic field acting at a specific anatomical location. We are currently working to better understand the relationship between magnetic field strength and localized cell retention. A study is also underway to evaluate other cell types (e.g., MSCs) for this procedure as well as to assess the functional efficacy of this approach. This study will involve a therapeutic model of neurodegenerative disease, where currently the clinical standard of care for cellular transplantation involves the highly invasive method of direct needle injections with the associated risks (e.g., bleeding and infection) and insufficient distribution. The ability to noninvasively deliver exogenously administered cells in an efficient and targeted manner at therapeutically efficacious levels could potentially make cellular therapy more attractive for a variety of existing applications. Demonstrating that the procedure is safe and effective may also provide incentives to propose and develop additional treatments for the ever-emerging field of cellular therapy.
